# The ability of Interleukin–10 to negate haemozoin-related pro-inflammatory effects has the potential to restore impaired macrophage function associated with malaria infection

**DOI:** 10.1186/s12936-023-04539-w

**Published:** 2023-04-14

**Authors:** Dumizulu Tembo, Visopo Harawa, Tam C. Tran, Louise Afran, Malcolm E. Molyneux, Terrie E. Taylor, Karl B. Seydel, Tonney Nyirenda, David G. Russell, Wilson Mandala

**Affiliations:** 1 Malawi-Liverpool-Wellcome Trust Clinical Research Programme, Blantyre, Malawi; 2 Department of Microbiology and Immunology, College of Veterinary Medicine, Cornell University, Ithaca, NY USA; 3 Liverpool School of Tropical Medicine, Liverpool, UK; 4 University of Liverpool, Liverpool, UK; 5 Blantyre Malaria Project, Blantyre, Malawi; 6 Michigan State University, Michigan, USA; 7 Kamuzu University of Health Sciences, Blantyre, Malawi; 8 Acadamey of Medical Sciences, Malawi University of Science and Technology, Blantyre, Malawi

**Keywords:** Malaria, Cytokines, Macrophages, Hemozoin

## Abstract

**Background:**

Although pro-inflammatory cytokines are involved in the clearance of *Plasmodium falciparum* during the early stages of the infection, increased levels of these cytokines have been implicated in the pathogenesis of severe malaria. Amongst various parasite-derived inducers of inflammation, the malarial pigment haemozoin (Hz), which accumulates in monocytes, macrophages and other immune cells during infection, has been shown to significantly contribute to dysregulation of the normal inflammatory cascades.

**Methods:**

The direct effect of Hz-loading on cytokine production by monocytes and the indirect effect of Hz on cytokine production by myeloid cells was investigated during acute malaria and convalescence using archived plasma samples from studies investigating *P. falciparum* malaria pathogenesis in Malawian subjects. Further, the possible inhibitory effect of IL-10 on Hz-loaded cells was examined, and the proportion of cytokine-producing T-cells and monocytes during acute malaria and in convalescence was characterized.

**Results:**

Hz contributed towards an increase in the production of inflammatory cytokines, such as Interferon Gamma (IFN-γ), Tumor Necrosis Factor (TNF) and Interleukin 2 (IL-2) by various cells. In contrast, the cytokine IL-10 was observed to have a dose-dependent suppressive effect on the production of TNF among other cytokines. Cerebral malaria (CM) was characterized by impaired monocyte functions, which normalized in convalescence. CM was also characterized by reduced levels of IFN-γ-producing T cell subsets, and reduced expression of immune recognition receptors HLA-DR and CD 86, which also normalized in convalescence. However, CM and other clinical malaria groups were characterized by significantly higher plasma levels of pro-inflammatory cytokines than healthy controls, implicating anti-inflammatory cytokines in balancing the immune response.

**Conclusions:**

Acute CM was characterized by elevated plasma levels of pro-inflammatory cytokines and chemokines but lower proportions of cytokine-producing T-cells and monocytes that normalize during convalescence. IL-10 is also shown to have the potential to indirectly prevent excessive inflammation. Cytokine production dysregulated by the accumulation of Hz appears to impair the balance of the immune response to malaria and exacerbates pathology.

**Supplementary Information:**

The online version contains supplementary material available at 10.1186/s12936-023-04539-w.

## Background

Despite gains achieved in the fight against malaria through different malaria control measures such as the use of insecticide-treated nets (ITNs) and indoor residual spraying (IRS), malaria still contributes substantially towards child mortality, causing well over 600,000 deaths every year globally [1]. The outcome of *Plasmodium falciparum* infection, the most prevalent and pathogenic malaria parasite, varies with the age of a person, their immune status linked to prior exposure, and parasite genetic diversity [2]. The roles played by both the innate and adaptive immune systems in the pathogenesis of malaria have been studied extensively.

Studies in both adults and children [3–6] suggest inflammation in malaria is a bimodal entity. Systemic elevations of pro-inflammatory cytokines such as TNF, IFN-γ, IL-2, IL-6 and IL-12p70 and of chemokines like MIG/CXCL9 during the acute infection phase contribute towards parasite clearance [7]. However, excess and unregulated production of these cytokines and chemokines is associated with the development of severe disease and is linked to other complications such as multiple organ system failures and mortality [8]. In the second phase of the infection, once parasite clearance has been achieved, anti-inflammatory mediators, such as transforming growth factor beta (TGF-β) and IL-10 are released to reduce ongoing inflammation. Overall this suggests that phase-specific balance between pro- and anti-inflammatory cytokines is critical to control infection whilst minimising immunopathology [9]. However, the regulatory circuits responsible for this homeostasis remain poorly understood.

Although elevated levels of cytokines like IL-6, IL-1β and IL-8 characterize acute CM [10] and correlate with disease severity in both adults and children [11–13], elevated levels of IL-10 and TNF are characteristic of severe malarial anaemia (SMA) and high parasitaemia in young African children [14–17]. IL-10 is produced by both CD4 + T cells and B cells and is capable of blocking the production of other cytokines such as IL-1β, IL-6 and TNF [18, 19]. In addition, by enhancing the proliferation of B cells, IL-10 contributes towards the humoral response to malaria [19, 20].

IL-10 achieves blockage of cytokine and chemokine production by targeting monocytes and macrophages and this in turn dampens inflammation through the down-regulation of the expression of MHC class II and co-stimulatory molecules on antigen-presenting cells [20]. As expected, low IL-10 levels usually result in an increase in TNFlevels which is then followed by an increase in IFN-γ production. Secondly, any impairment in the function of the HLA-DR isotype expressed on monocytes could essentially result in defective immune responses towards various pathogens including *P. falciparum* parasites. Evidence for this hypothesis is the observation that low levels of HLA-DR expression in patients with sepsis have been associated with poor recovery and mortality rate [21–23]. Therefore, a better understanding of the functional mechanisms of macrophages and monocytes could add to the knowledge of the precarious balance between pro- and anti-inflammatory processes.

During intra-erythrocytic development, *P. falciparum* invades red blood cells (RBCs) and digests haemoglobin (Hb). This results in the production of various metabolites including Hz, which is formed and sequestered within the digestive vacuole of parasitized RBCs (pRBCs) [24]. Hz is released together with merozoites upon rupture of pRBCs and is found in increased concentrations in the peripheral circulation where it is engulfed by various cells such as macrophages, monocytes and neutrophils [25, 26]. Although Hz is known to have an effect on the function of the affected cells during earlier stages of malaria infection, during the late acute stages of malaria infection, Hz seems to favour the production of cytokines and chemokines [27, 28]. However, when Hz persists within the affected phagocytic cells it can lead to malaria-related immune suppression [29–32], which affects antigen presentation by the affected cells [32, 33], impairs phagocytosis [34, 35], inhibits the generation of oxidative burst [31], and also the production of nitric oxide (NO) [35]. While data from tissue culture models implicate Hz as a major contributing factor towards immune dysregulation associated with malaria, little has been done to link the significance of Hz to the evolving immune response during in vivo infection. This study assessed how Hz affects the function of monocytes and macrophages during *P. falciparum* malaria, and to explore the ability of IL-10 to modulate this effect. Further, the cytokine profiles previously linked to different clinical forms of P. falciparum malaria in Malawian children were characterized.

## Methods

### Ethical approval

Human specimens and data were obtained from two separate studies. Data on the Hz-loaded monocytes, intracellular cytokine staining and expression of monocyte surface markers were obtained from a study that recruited Malawian children presenting with different clinical malaria groups, either uncomplicated malaria (UM), severe malaria anaemia (SMA) or cerebral malaria (CM) from November 2005 to February 2007. These were patients admitted to the Paediatric Research Ward at Queen Elizabeth Central Hospital (QECH) in Blantyre Malawi. Ethical approval for that study was obtained from the College of Medicine Research and Ethics Committee [COMREC—Protocol Number P.01/02/176].

Samples and data on the cytokine Luminex work were obtained from malaria patients admitted at the Paediatric Research Ward at Queen Elizabeth Central Hospital (QECH) in Blantyre Malawi from January 2013 to December 2016. Forty healthy children without a previous history of malaria or parasitaemia recruited at Ndirande Health Centre in Blantyre during routine attendance of their vaccination appointment were included as controls. The ethical approval for this second study was also obtained from the College of Medicine Research Ethics Committee [Protocol number P.08/15/1785]. Written informed consent was obtained from parents or guardians of participating children in both studies.

### Study population

Blood samples were collected from two cohorts of paediatric participants. The first cohort was of paediatric patients presenting with either UM, SMA, or CM admitted at the Paediatric Research Ward at QECH between 2005 and 2007 under a clinicopathologic study the details of which have been described before [44, 59, 62]. For the malaria cases, each potential study participant was examined by a research nurse and/or clinical officer. Patients between the ages of 5 months to 12 years diagnosed with *P. falciparum* following examination of their thick blood smear were enrolled and a 5 ml venous blood sample was obtained.

Study participants who presented with CM had a Blantyre Coma Score (BCS) of two or less at admission and four hours later, while participants in all other malaria groups (SMA and UM) had a score of five at both time points. A funduscopic examination was also performed on all patients to determine the presence or absence of malaria retinopathy [36] and was further categorized as either being Retinopathy positive (Ret +) or Retinopathy negative (Ret-) based on the funduscopic examination.

Study participants with severe malarial anaemia (SMA) and a blood haemoglobin concentration of 5 g/dL or less, whereas study participants in the other groups had a haemoglobin concentration above this level. All participants who tested positive for HIV infection during the screening stage were excluded from the study and were immediately referred to the antiretroviral therapy clinic. Potential study participants were also excluded if they had a positive test for bacterial infection in cerebral spinal fluid (CSF) culture.

The healthy controls from this cohort were medically-well children between the ages of 6 months and 5 years, who had not been infected with symptomatic malaria in the past four months and who were attending surgical outpatient clinics at QECH and Beit Cure International Hospital in Blantyre. In total, 196 children were prospectively enrolled into the four groups namely CM, SMA, UM, and healthy controls.

The second cohort used in Luminex cytokine analysis comprised CM retinopathy-positive children (n = 54) and healthy controls (n = 40) aged between 6 months and 5 years recruited between 2013 and 2016 whose details are provided in Additional file 2: Table S1. The healthy controls were recruited during routine attendance of their vaccination appointment. In total 94 children were recruited under this cohort. In addition, fresh blood was collected from seven adults (> 25 years) who had no history of malaria in the past year and had provided written consent to have a 10 mL venous blood sample collected for the in vitro whole blood assays. Although all laboratory analyses that required the use of fresh whole blood were done within 8 h from the time of blood sample collection, plasma samples were stored at − 80 °C until the day of analysis.

### Haemozoin isolation and quantification

Laboratory strain 3D7 *P. falciparum* was cultured under sterile conditions. Briefly, parasites were maintained in vitro at 5% haematocrit with RPMI 1640 supplemented with L-glutamine, 1 M HEPES, 7.5% NaHCO_3_, gentamycin 50 mg/ml, 200 mM hypoxanthine and 0.5% Albumax II (Life Technologies). Once high parasitaemia was reached, the parasites were left for one or more cycles to allow sufficient natural egress of merozoites and release of Hz. The cultures were spun at 400 × g for 5 min. The supernatants were collected and run through an LS Miltenyi magnetic column. Columns were washed with 3 ml of 5% FBS in PBS or RPMI and eluted. Hz was harvested by centrifuging for 5 min at 3500 × g and re-suspended in 500 µL 5% FBS in PBS or RPMI. Hz was quantified as previously described [37]. Briefly, Hz was incubated in 2% SDS/20 mM sodium hydroxide for 1 h and the solubilized monomeric haem polymer was quantified using a spectrophotometer at 400 nm.

### Determination of the effect of patient plasma IL-10 on TNF production

Malaria patient plasma may contain multiple cytokines and other factors exhibiting anti-inflammatory properties. IL-10 is one of the best characterized anti-inflammatory cytokines and was used as a potential indicator for anti-inflammatory properties to assess its possible activity in controlling Hz-induced inflammation of the human disease. Based on this supposition, patient plasma containing different amounts of IL-10 was selected. Plasma samples were selected based on availability. A total of 15 samples were identified for this purpose with IL-10 concentrations > 700 pg/mL to < 200 pg/ml. Blood was collected from malaria-naïve healthy adults in sodium heparin tubes and spun at 700 × g for 10 min to separate plasma. 50µL of the pelleted cells was aliquoted into 96-well plates. An equal volume of patient plasma was introduced, and Hz was added to each experimental well at a final concentration of 60 nmol/mL. Half of the experimental wells were also stimulated with human IL-10 recombinant protein (R&D Systems) at a final concentration of 0.75 ng/mL as per the manufacturer’s instructions. Control wells were stimulated with Hz only. The plate was incubated at 37 °C in a CO_2_ incubator for 24 h, spun down and the plasma supernatants were harvested and kept at − 80 °C for TNF analysis by Enzyme-Linked Immunosorbent Assay (ELISA).

### Measuring the effects of haemozoin on cytokine production by immune cells

Whole blood samples from healthy donors were diluted with an equal volume of HEPES-buffered RPMI and kept at 37 °C until required. Hz was added at a final concentration of 60 nmol/mL for 250 µL diluted blood. The tubes were kept at 37 °C and supernatants were harvested at times 0, 1, 2, 4, 6, 12, 18 and 24-h time points for cytokine analysis using Luminex as previously described using 13 analytes; GM-CSF, IFN-γ, IL-1β, IL-2, IL-4, IL-5, IL-6, IL-7, IL-8, IL-10, IL-12 (p70), IL-13, and TNF kit (Millipore).

### Electron microscopy

Samples were fixed and processed for electron microscopy as detailed previously [38].

### Determination of the proportion of haemozoin-loaded monocytes

Malaria parasitaemia was determined by thick and thin blood films. Reading of the slides for malaria parasites and Hz was performed according to standard procedures [39]. In brief, thick malaria blood smear slides were prepared by staining with 2% Giemsa for 15 min. After drying, the haemozoin-loaded monocytes were counted. Expression of HLA-DR and CD86 as primary markers to assess the activation status of antigen-presenting cells (APC), particularly focusing on monocytes was investigated.

For each sample, 25μλ of EDTA anti-coagulated blood was mixed with 1μλ of three directly-conjugated antibodies: a FITC-conjugated antibody anti-HLA-DR [L243] and FITC-conjugated anti-CD86 [2331 (FUN-1)] and anti-CD14-APC [MΦP9] (all antibodies details are provided in Additional file 2: Table S2). Samples were incubated for 15 min in the dark at room temperature. 500μL of 1xFACS lysing solution (Becton Dickinson) was added to each tube and incubated in the dark for 10 min at room temperature. Cells were washed twice with 2 mL of PBS and fixed with 100μλ PBS/1% formaldehyde. Data were acquired on a BD FACSCalibur flow cytometer and analysed using CellQuest Pro software.

### Detection of TNF-α and IL-6-producing monocytes

One mL of heparinized patient blood was mixed with 10μL of 100μγ/mL LPS and 10μL of 1μγ/mL Brefeldin A (BFA). The mixture was vortexed and incubated at 37 °C, 5% CO_2_ for four hours in loose-capped tubes. A negative control from a healthy study participant was stimulated under the same conditions. An aliquot of 50μL of stimulated and unstimulated blood was labelled with 2μL of anti-CD14-APC [MΦP9] and incubated in the dark for 15 min. 2 mL of FACS lysis solution was added to each tube, vortexed and incubated in the dark for 10 min. Tubes were centrifuged at 500 × g and 4 °C for 5 min, the supernatant aspirated and 500 μL of FACS Permeabilizing solution (Becton Dickinson) was added to each tube before being incubated in the dark for 10 min. Cells were then washed with 2 mL of PBS/0.5% Bovine Serum Albumin (BSA). 4 μL of PE-conjugated anti-TNF [6401.111] or anti-IL-6 [MQ2-13A5] were added to each tube, vortexed and incubated for 30 min in the dark. Tubes were then washed with 2 mL of PBS/0.5% BSA and the cells were fixed with 200 μL PBS/1% formaldehyde. All incubation sets were performed at room temperature. Data were acquired and analysed on a FACSCalibur instrument within an hour.

### Detection of IFN-γ and TNF-α-producing T cells

These assays were done on fresh blood samples within eight hours of collection when the first cohort was recruited between 2005 and 2007. The *ex-vivo* cytokine expression of T cells (CD3 +) was examined in the different malaria groups. Also, the functionality of the malaria-specific T cells (CD3 +) was measured in whole blood stimulated with *P. falciparum* schizonts lysate and phorbol myristate acetate (PMA) and ionomycin as positive control by flow cytometry based on their production of IFN-γ and TNF. One mL heparinized blood was pipetted into a 15 mL polypropylene tube to which 10µL of the pooled schizont lysate and 5 µL of anti-CD28 were added. The mixture was vortexed and incubated for 3 h at 37 °C with 5% CO_2_, with the cap loosely closed. 10µL of diluted BFA was added to the tube at a final concentration of 1 µg/mL and the mixture vortexed again before being incubated for an extra 2 h. 2 μL of monoclonal antibodies against lymphocyte surface markers (anti-CD3-PerCP [SK7], anti-CD4-FITC [L120] and anti-CD8-FITC [SK1], all from Becton Dickinson) were used to differentiate lymphocyte subpopulations. 50µL of the specific stimulated or unstimulated blood sample was used per tube and mixed with the antibodies.

The tubes were incubated in the dark at room temperature for 15 min. RBCs were lysed with 2 mL of 1 × FACS lysis solution (Becton Dickinson) in the dark for 10 min. The tubes were vortexed and centrifuged at 500 × g at 4 °C for 5 min. The supernatant was aspirated and tubes were vortexed before 500 µL of 1 × FACS Perm (Becton Dickinson) was added to each tube and incubated in the dark for 10 min. 1.5 mL of PBS containing 0.5% Bovine Serum Albumin (BSA) (Aldrich) was added to each tube, briefly vortexed and centrifuged at 500 × g at 4 °C for 5 min. The supernatant was aspirated and the tubes vortexed. 4µL of PE-conjugated anti-IFN-γ [25723.11] and anti-TNF [6401.111] antibodies were added to each tube and 2 µL of human IgG (Sigma) was added to tubes for labelling blood stimulated with malaria schizonts lysate as a blocking reagent to reduce unspecific background binding of the antibodies.

Each tube was vortexed and incubated for 30 min in the dark at room temperature. 2 mL of PBS/0.5% BSA was added to each tube, vortexed and centrifuged at 700 × g at 4 °C for 5 min. The supernatant was aspirated and the tubes vortexed before the cells were fixed with 200 µL of PBS/1% formaldehyde solution. PMA (Aldrich) and ionomycin were used as positive control by adding to a 1 ml mixture of blood and RPMI-1640 at a final concentration of 10 ng/mL and 1 µg/mL, respectively. Samples were acquired by Flow Cytometer within an hour of being stained and fixed.

### Determination of activated macrophages

Similarly, these assays were also conducted on fresh blood samples within eight hours of collection when the first paediatric cohort was recruited between 2005 and 2007. All incubations were performed at room temperature unless otherwise specified. For each sample, 25 μL of EDTA study patient blood was mixed with 1 μL of three directly-conjugated antibodies: a FITC-conjugated antibody of either anti-HLA-DR [L243] or anti-CD86 [2331 (FUN-1)] and anti-CD14-APC. Samples were incubated for 15 min in the dark. 500 μL of FACS lysing solution (Becton Dickinson) was added to each tube and incubated in the dark for 10 min. Cells were washed twice with 2 mL of PBS and fixed with 100 μL PBS/1% formaldehyde. Data were acquired on a BD FACSCalibur flow cytometer and analysed using CellQuest Pro software.

### Measurement of plasma cytokine levels by Luminex

Plasma samples archived from the first and second paediatric cohorts were used for these assays. For plasma extraction, whole blood was aseptically collected into sodium heparin Vacutainer^®^ tubes (Becton Dickson and Company, USA), and was centrifuged at 700 × g for 10 min at room temperature. The plasma was separated and stored at -80 °C until use. Luminex technology (Luminex Corporation, Austin, TX, USA) was used to measure the 13 plasma analytes previously described according to the manufacturer's instructions. Briefly, 25 μL of sample and kit standards were incubated with 25 μL of the mixed microbeads overnight in a 96-well plate. After washing the plate three times, 25 μL of biotin antibody was added to each well and incubated at room temperature for 60 min. After washing, 25 μL of Streptavidin-PE was added to each well and incubated for 30 min at room temperature. The microbeads were suspended in 150 μL of reading buffer. The plate was read on a Luminex MAGPIX multiplex reader and data were analysed using xPONENT software (v4.2). Samples below the limit of detection were either given a value at the limit of detection or half the limit of detection when log-transformed for statistical analysis.

### Measurement of plasma cytokine levels by ELISA

Similarly, plasma samples from the two cohorts were used for this set of experiments. TNF levels in the plasma were determined by ELISA from a single-analyte ELISArray TNF (Qiagen, USA) kit according to the manufacturer's instructions. All incubations were performed at room temperature unless otherwise specified. Briefly, 50 µL of standards and samples were added to appropriate wells of TNF pre-coated plates and incubated for 2 h. 100µL of detection antibody was added after washing three times and incubated for an hour. After washing three times, 100 µL of Avidin-Horseradish Peroxidase (HRP) was added and incubated for 30 min. The plates were washed four times and incubated with 100 µL development solution for 15 min. 100 µL of stop solution was added and plates were read at 450 nm on a SpectraMax M2 (Molecular Devices) and analysed using SoftMax Pro (v) software to fit the kit’s standards curve. Background absorbance was subtracted from sample measurements.

### Statistical analysis

Statistical analyses and graphical presentations were performed using GraphPad Prism 5 (GraphPad Software, USA). Pearson’s χ^2^ test was used for between-group comparisons of dichotomous variables. For statistical analysis of plasma cytokine and chemokine data, statistical differences between groups were determined after performing prior logarithmic transformation of the data. The Inter-group comparisons were performed using the Kruskal–Wallis test. Unpaired data from patients were evaluated using the Wilcoxon rank sum test. Associations were analysed using the Spearman test and differences were considered statistically significant when p < 0.05.

## Results

### Demographic details of the study participants

For the first paediatric cohort, following exclusions, 196 children aged between 6 months and 7 years were recruited (n = 66 for UM, n = 42 for SMA, n = 36 for CM, n = 52 for healthy control). Of the children with malaria, 73 (n = 34 for UM, n = 21 for SMA and n = 18 for CM) were successfully followed up a month after treatment. Data regarding malaria incidence, parasite density, and haemoglobin levels at the time of blood sampling are shown in Table 1. In summary, 121 (78%) were male and 5 (3.25%) study participants died after being enrolled into the study before they could be followed up. The second paediatric cohort of CM retinopathy-positive children (n = 54) and healthy controls (n = 40) aged between 6 months and 5 years were recruited.

**Table 1:** Details of the study participants for the different clinical malaria types and in healthy controls in the first paediatric cohort recruited between 2005 and 2007

Clinical group	Healthy controls	Uncomplicated malaria	Severe malaria anemia	Cerebral malaria
Number	52	66	42	36
Death after admission	–	0	1	4
Reviewed in convalescence	–	40	25	22
Sex (M:F)	35:14	45:21	27:15	14:22
Age (months)	20 (5–76)	27 (6–58)	23 (5–38)	30 (5–84)
Parasitaemia (parasite/µL)	0	52,300 (460—768,000)	3,500 (20–296,000)	41,800 (900–517,000)
Blantyre coma score	5	5	5	1 (0–2)
Hemoglobin (g/dL)	11.2 (7.0–14.1)	9.3 (5.0–13.0)	3.9 (2.4–4.9)	7.7 (5.3–12.5)

Values are medians and range in brackets

### Pro-inflammatory cytokines are higher in children with cerebral malaria

**Fig. 1:**
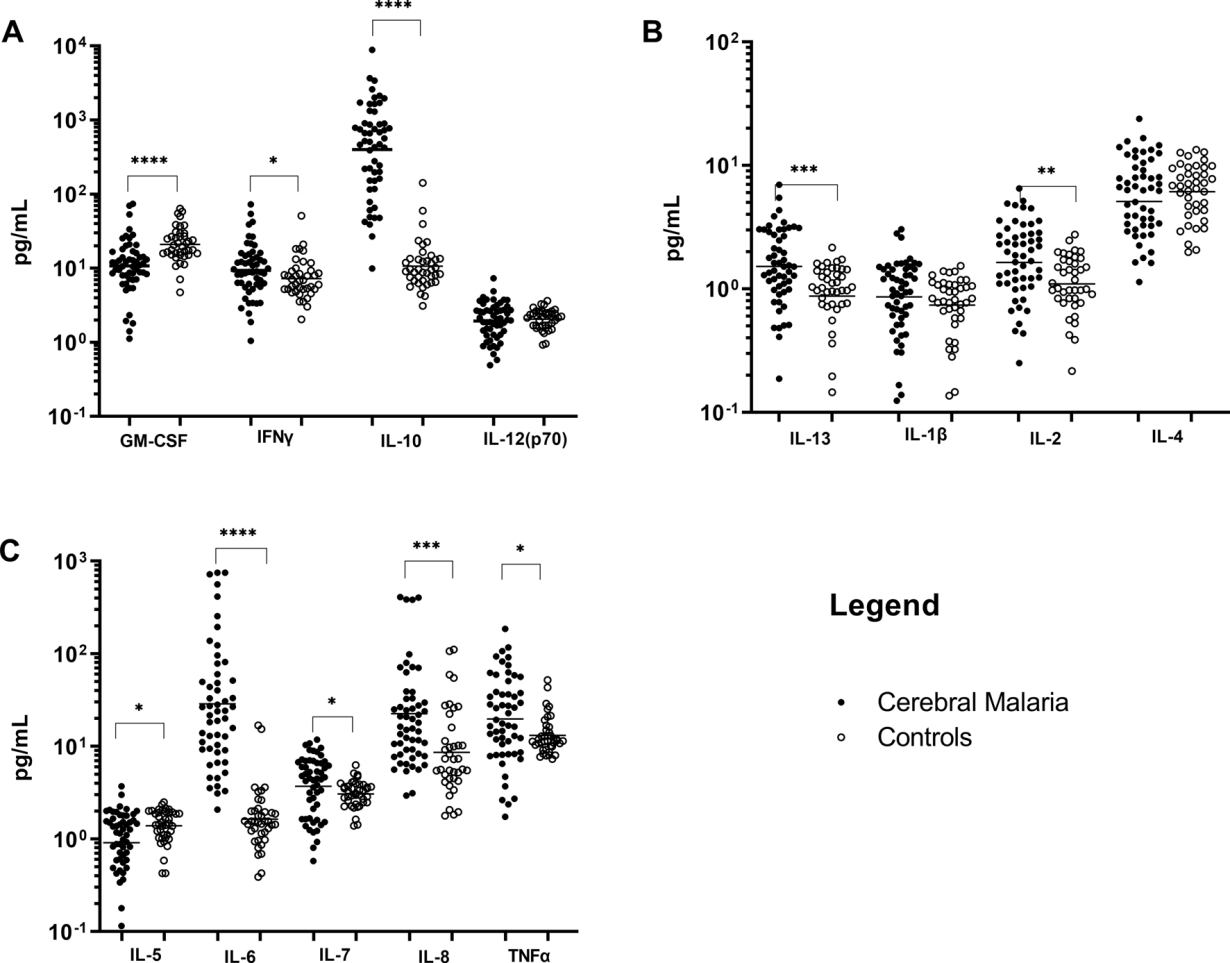
Scatter plots of 13 cytokines levels in plasma of patients presenting with retinopathy positive cerebral malaria (black dots) and of healthy controls (hollow dots): Each dot corresponds to an individual participant. The black bars denote geometric mean. The significance of the differences is calculated by Mann–Whitney test and are indicated by asterisks as follows: *, p ≤ 0.05; **, p ≤ 0.001; ***, p ≤ 0.0001; ****, p < 0.0001

Plasma cytokine concentrations (pg/mL) were measured using a multiplex bead array on a Luminex platform. Distinct differences were observed between children presenting with CM and the healthy children with the analysis showing the following cytokines IFN-γ, IL-2, IL-6, IL-7, IL-8, IL-10, IL-13, and TNF and being significantly higher in CM patients than in controls (*P* < 0.05) (Fig. 1A, C).

In contrast, the plasma levels of GM-CSF and IL-5 were significantly higher in healthy controls compared to the levels observed in CM patients (*P* < 0.05). Several cytokines, namely IFN-γ, IL-6, TNF, IL-8, and IL-10 have already been shown to be elevated during acute malaria infections, regardless of severity, in studies involving both adults and children [4–6, 11, 13, 40]. Furthermore, the cytokines TNF and IL-10 have also been shown to distinguish between severe malaria anaemia (SMA) and high parasitaemia in African children [14, 15].

### Experimental platforms for assessing the impact of Hz on macrophage functions

**Fig. 2:**
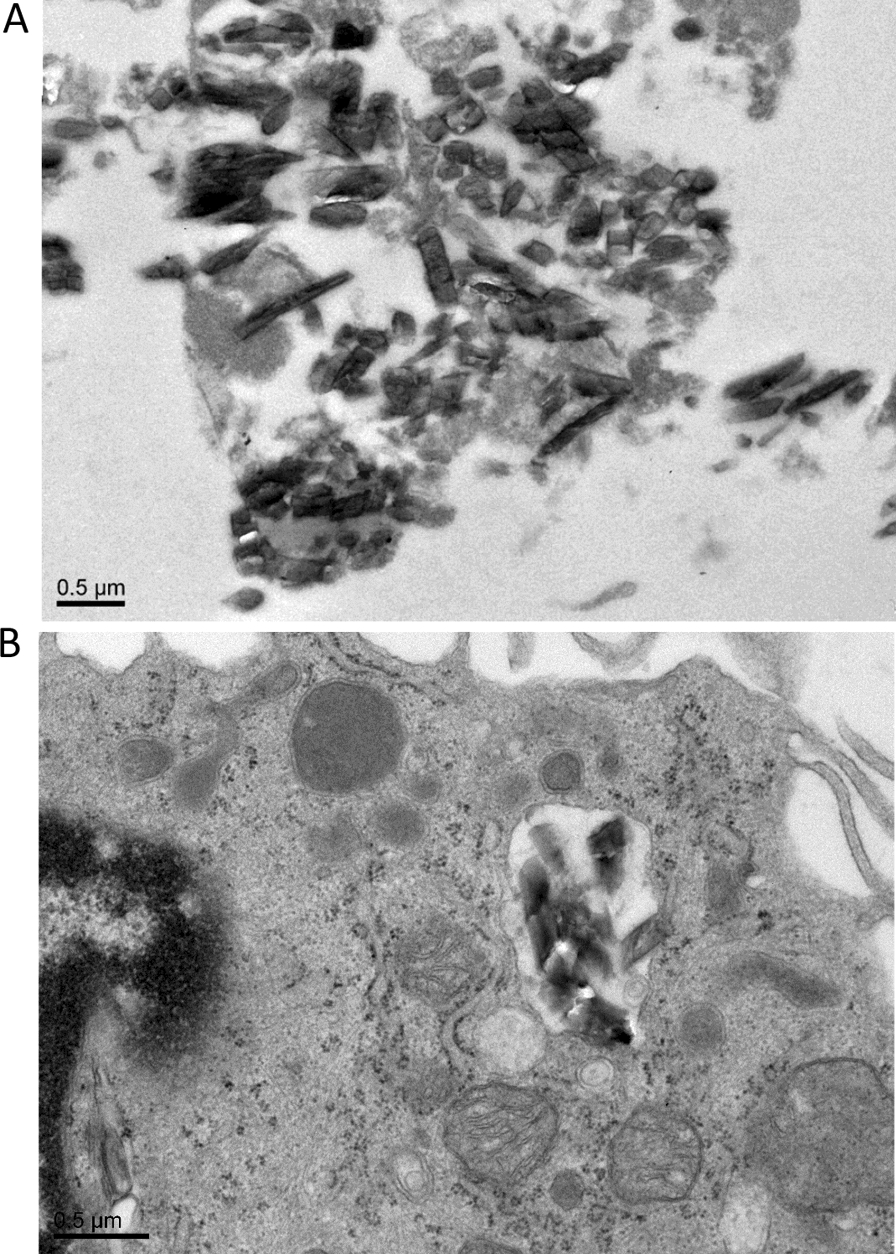
Electronic microscopy images of haemozoin after magnetic isolation (**A**) and localized within the human macrophage after 4 h incubation with haemozoin (**B**)

**Fig. 3:**
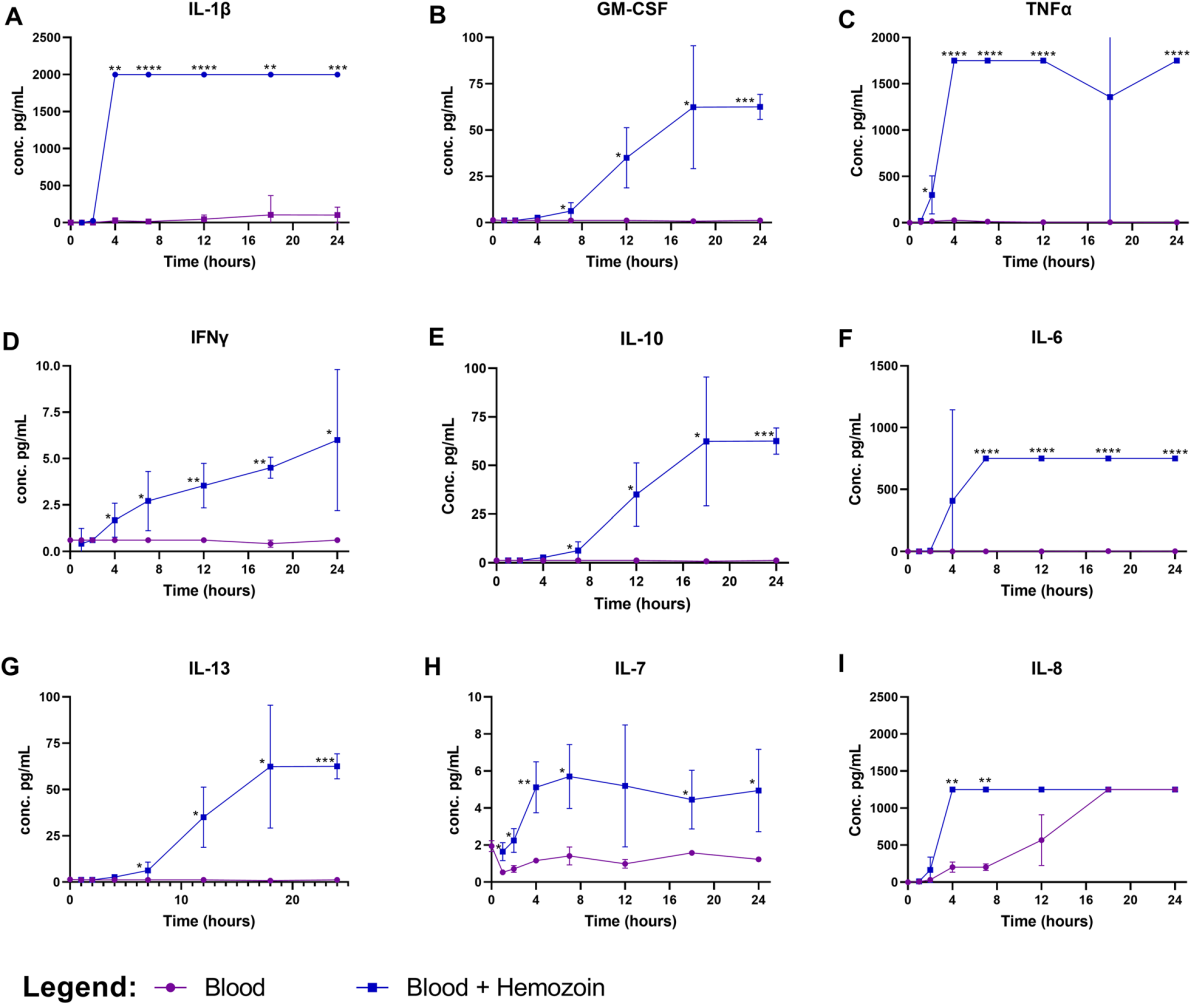
The effect of haemozoin on cytokine production in vitro*:* Diluted whole blood from healthy volunteers were stimulated with haemozoin at a final concentration of 60 nmol/mL at 37 °C. Supernatants were collected at 4, 8, 12, 16, 20 and 24 h. Cytokines IL-1β (**A**), GM-CSF (**B**), TNF (**C**), IFN-γ (**D**), IL-10 (**E**), IL-6 (**F**), IL-13 (**G**), IL-7 (**H**), and IL8 (**I**) are measured over time. The 95% confidence interval for each cytokine is reported. Asterisks show significant differences found between unstimulated blood (purple) and blood stimulated with haemozoin (blue) with multiple comparison t-test. *, p ≤ 0.05; **, p ≤ 0.001; ***, p ≤ 0.0001; ****, p < 0.0001

There is some debate over the inflammatory moieties in “artificial” Hz, which is stripped of the lipids and proteins that are considered to drive phagocytosis and inflammation [41, 42]. Therefore, Hz was isolated directly from the *P. falciparum* culture medium, post-rupture of RBCs, by magnetic enrichment, (Fig. 2). The Hz fraction was fed to human monocytes derived macrophages from healthy donors, which readily phagocytized the parasite pigment. The response to this stimulation was assessed using both Luminex analysis against a panel of inflammatory cytokines, and by several functional assays, such as phagocytosis. The response to Hz was tested in a human, whole blood assay where the induction of cytokines was measured as a function of time following the addition of Hz for 1, 2, 4, 8, 12, 16, 18 and 24 h. The following 13 cytokines; IFN-γ, IL-12, IL-13, IL-1β, IL-4, IL-5, IL-6, TNF, IL-2, IL-7, IL-8, IL-10 and GM-CSF were measured using Luminex technologies. Some panels of cytokines, such as TNF and IL-1β were rapidly produced, while others were produced slightly slower following stimulation. Overall, the majority of the cytokines; IL-1β, GM-CSF, TNF, IFN-γ, IL-10, IL-6, IL-13 and IL-7 gradually increased over time (Fig. 3A–H). Eight of these cytokines (IL-1β, TNF, IL-10, IL-13, IL-7, IL-6, IFN-γ and IL-8) were also found significantly higher in the plasma of CM compared to the controls as shown in Fig. 1. While IL-4 and IL-12 amounts were not different between CM and controls (Fig. 1A and B), they were also not statistically different between Hz stimulated and unstimulated conditions.

### Haemozoin impairs monocyte function

**Fig. 4:**
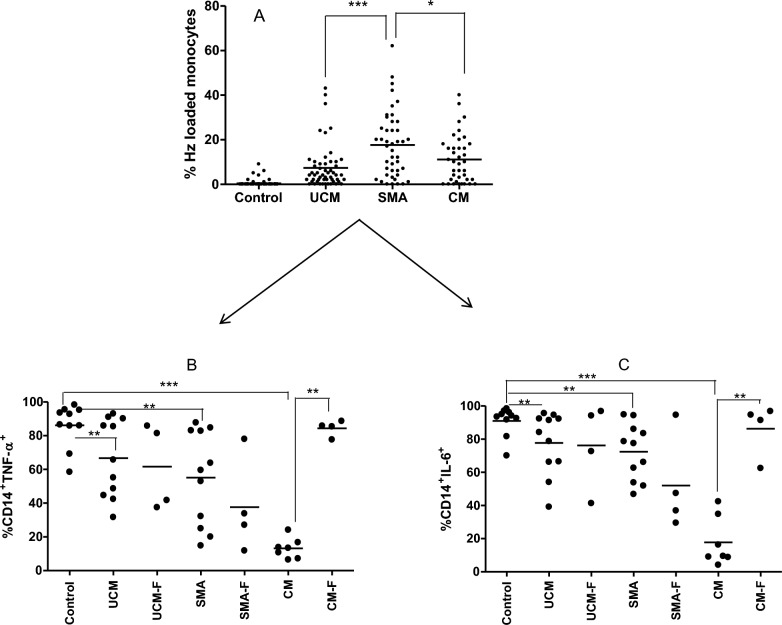
Monocytes exhibit an impairment in cytokine production for malaria patients, which correlated with the presence of haemozoin: Haemozoin-loaded monocytes were counted by microscopy from thick malaria smear stained with 2% Giemsa. Median proportion of haemozoin-loaded monocytes in controls and acute malaria types were recorded (4**A**). An intracellular cytokine staining was performed on 50 µL blood from healthy controls and study participants with different malaria types (UM: uncomplicated malaria; SMA: severe malaria anaemia; CM cerebral malaria) during acute infection and 30 days in convalescence (UM-F, SMA-F and CM-F). The blood was initially labelled with antibodies against CD14. Four hours post brefeldin A blocking and red blood cells lysing, the cells were stained with antibodies against TNF and IL-6. Each dot corresponds to an individual participant. Statistical significance was determined from the medians (10th and 90th percentiles) of proportions of TNF-producing monocytes (4**B**) and IL-6 producing monocytes (4**C**) analysed by flow cytometry. **** = p < 0.0001, *** = p < 0.001, ** = p < 0.01, * = p < 0.05

The effect of Hz on the function of monocytes was examined in the three malaria groups UCM, SMA and CM, and the healthy control group. The percentage of Hz-loaded monocytes was determined in each malaria group by microscopy on thick malaria blood smear slides. Acute SMA cases had the highest levels of Hz-loaded monocytes at 20% compared to the UCM at 9% (p < 0.0001) and 12% in CM (p < 0.05) (Fig. 4A). All malaria groups had significantly lower TNF and IL-6-producing CD14 + monocytes during the acute stage when compared to the levels observed in the healthy controls (Fig. 4B and C; the representative flow cytometry plots are described in Additional file 1: Fig. S1). However, of the three malaria groups, CM had the lowest proportion of cytokine-producing monocytes compared to the levels observed in healthy controls (Fig. 4B and C). For UCM and SMA, the proportion of TNF and IL-6-producing monocytes during convalescence remained significantly lower than in the healthy controls (p < 0.01). However, in convalescent CM the proportion of these TNF-α and IL-6-producing monocytes increased significantly (p < 0.0001 compared to acute CM) compared to those observed during acute infection and were similar to those observed in healthy control level, suggesting that monocyte-cytokine producing ability “normalizes” in convalescent CM compare to SMA.

### The inhibitory effect of IL-10 on haemozoin-induced TNF production

**Fig. 5:**
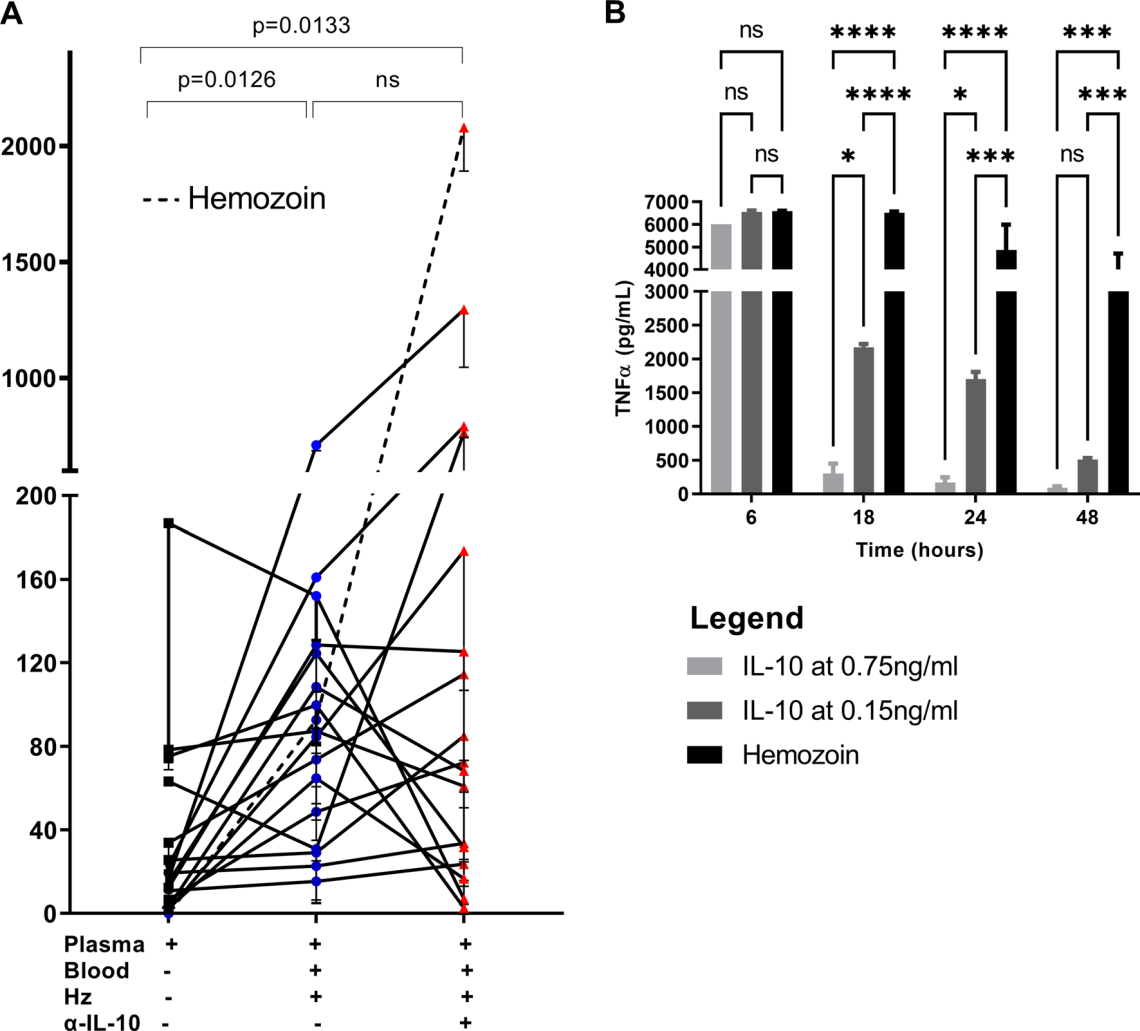
The IL10 decreases the production of TNF in whole blood from malaria naïve stimulated with haemozoin: Whole blood from a healthy malaria naïve donor, and Hz was added at a final concentration of 60 nmol/mL. An equal volume of patient plasma was introduced, with IL-10 recombinant protein at a final concentration of 0.75 ng/mL added to half of the experimental wells. Control wells were stimulated with Hz only. After 24 h, supernatants were harvested and analysed for TNF by ELISA. The representative dot plots indicate the original concentration of TNF (pg/mL) contained in the patient plasma (black) is compared with TNF produced after stimulation with haemozoin (blue) and in the presence of anti-IL-10 antibody (red). Each dot corresponds to an individual participant (5**A**). Wells were stimulated in the presence or absence of two different concentrations of recombinant IL-10. After 18, 24, and 48 h, supernatants were harvested, analysed for TNF by ELISA. All comparisons were performed using Tukey from a permutation One-way analysis of variance (ANOVA). The mean values that are statistically significant are indicated by p < 0.05. In the bar graph, the bar shows the mean. Data represent the pool of 2 replicates. *P < 0.05; *** P < 0.001; **** P < 0.0001 (Two-way ANOVA multiple comparison); ns means differences are not significant

IL-10 is one of the well-characterized anti-inflammatory cytokines and was used as a potential indicator for anti-inflammatory properties to assess its possible activity in controlling Hz-induced inflammation of the malarial disease. Based on this supposition, patient plasma containing different amounts of IL-10 was selected, and TNF was used as an inflammation readout upon Hz simulation of whole blood from malaria naïve donors. Initially, the original TNF contained in each sample was measured (represented by the black squares in Fig. 5A) and used as the point of reference for subsequent TNF increase or decrease upon stimulation. Significantly high amounts of TNF were seen upon Hz stimulation (blue circles in Fig. 5A, compared to the original TNF (p = 0.0126). Also, TNF remains significantly higher even when the anti-IL-10 antibody was added (p = 0.0133). However, the overall TNF production upon Hz stimulation compared to the same condition plus anti-IL-10 antibody was insignificantly different (Fig. 5A). To gain further insights into the functional significance of IL-10 in Hz-induced inflammation, the amount of TNF produced upon adding varying concentrations of recombinant IL-10 to whole blood stimulated with Hz over 48 h was measured (Fig. 5B). The addition of IL-10 at 0.75 ng/mL significantly reduced TNF production compared to IL-10 added at 0.15 ng/mL. Overall, the addition of IL-10 to Hz-stimulated blood, regardless of concentration, significantly reduced TNF production at 18, 24, and 48 h compared to Hz-stimulated conditions where IL-10 was not added (Fig. 5B).

### Myeloid cells from CM patients are poorly activated

**Fig. 6:**
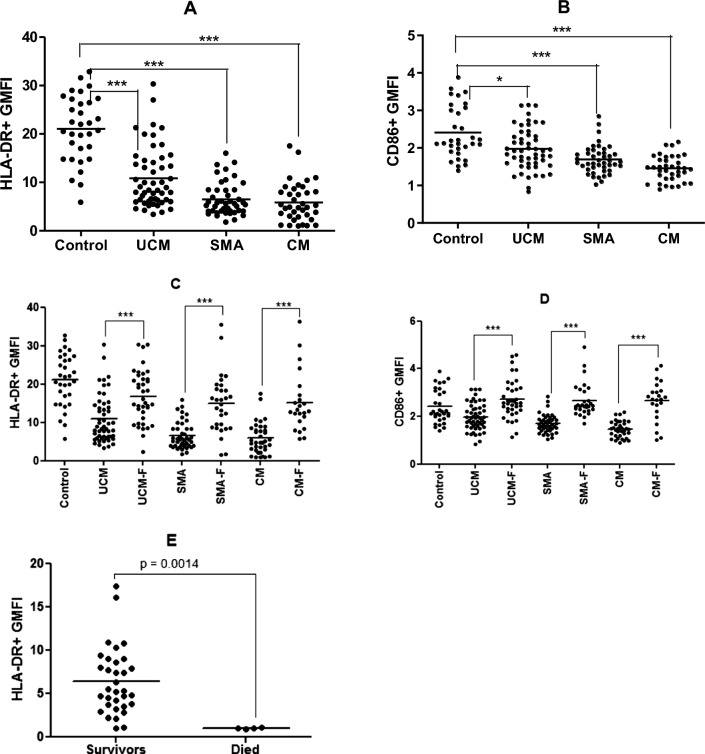
The level of activation of monocytes based on HLA-DR and CD86 expression: CD 14 positive monocytes of children presenting with acute uncomplicated malaria (UCM), severe malarial anaemia (SMA) or cerebral malaria (CM), and healthy aparasitaemic children (Control) were analysed during acute infection (4**A** and 4**B**) and convalescence (4**C** and 4**D**) by flow cytometry. HLA-DR expression is also compared between CM children who died compared to those who survived (**E**). Medians (10th & 90th percentiles) of geometric mean florescence intensity (GMFI) are reported. **** = p < 0.0001, *** = p < 0.001, ** = p < 0.01, * = p < 0.05

To determine the level of activation of the myeloid cells from patients in the different disease categories, the expression of HLA-DR and CD86 was examined in aparasitaemic controls and children presenting with different forms of malaria (UCM, SMA, and CM) during acute infection and during convalescence. The median percentage of peripheral blood monocytes expressing the HLA-DR antigen in healthy donors was 21.29 ± 1.2%. Likewise, the median geometric mean fluorescent intensity (GMFI) of CD86 expression by monocytes, expressed as GMFI, was 2.17 ± 0.13% in healthy controls. The representative flow cytometry histograms showing the gating for CD14-positive monocytes expressing HLA-DR and CD86 are described in Additional file 1: Fig. S2. The percentage of macrophages expressing HLA-DR and CD86 in the different malaria groups was significantly lower (Fig. 6A and B), suggesting ineffective or incomplete activation of the circulating macrophages compared to controls. This observation has been previously reported in severe malaria anaemia [43]. The majority of the macrophages in circulation may have already been exhausted due to sustained ingestion of Hz and other parasite debris. However, impairment is transient as a normal expression of these markers is observed in all convalescent groups (Fig. 6C and D). Although not significant, the expression of both markers decreases with disease severity, from UCM to CM. Figure 6E presents the comparison between the HLA-DR expression in CM cases who survived compared to the four who died. Low HLA-DR expression on monocytes is also characteristic of non-survivors of infection and sepsis [44].

### Impairment of T-cell function in falciparum malaria patients

**Fig. 7:**
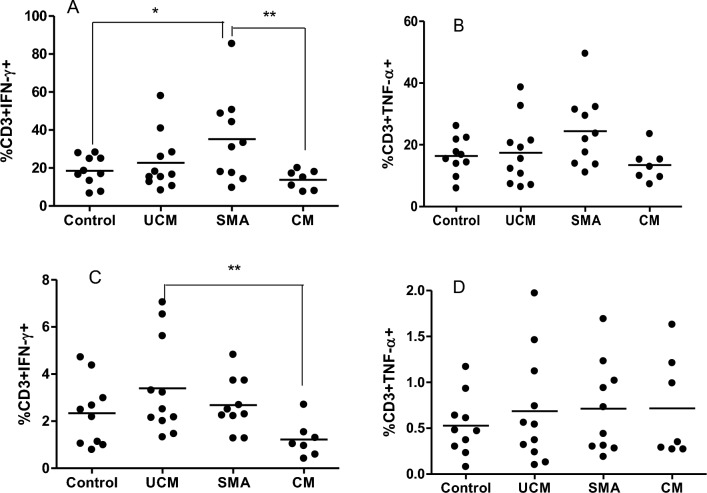
Impairment of malaria-specific T- cell function due to *P. falciparum*: IFN-γ and TNF producing CD3 + T cells were checked by flow cytometry for children with uncomplicated malaria (UCM), severe malarial anaemia (SMA) or cerebral malaria (CM), and healthy aparasitaemic children (Control). The cells were stimulated with phorbol myristate acetate/Ionomycin (7**A** and 7**B**) as a positive control, and *P. falciparum* schizonts lysate (7**C** and 7**D**) prior to cytokine measurements. IFN-γ- expressing CD3 + T cells (7**A** and 7**C**) and TNF-α- expressing CD3 + T cells (7**B** and 7**D**) in controls and different malaria types are reported as medians (10th and 90th percentiles). **** = p < 0.0001, *** = p < 0.001, ** = p < 0.01, * = p < 0.05

The median frequency of malaria-specific T cells responses producing IFN-γ after PMA/ION stimulation was higher in SMA at 40.8% (p < 0.05) versus 25% in the control group (Fig. 7A; the representative flow cytometry plots describing the gating strategy are in Additional file 1: Fig. S3). At 18.2%, the CM group produced less IFN-γ than SMA (p < 0.001). Upon stimulation with schizont lysate, a significant difference in IFN-γ production by T cells was only observed between UCM and CM, with the former group producing more at 3.6% (versus 1.4% for CM, p < 0.001; Fig. 7C). There were no significant differences in TNF production between the various groups upon stimulation with PMA + ION and schizonts lysate (Fig. 7B and D).

## Discussion

This study was aimed at further investigating the role of haemozoin on malaria pathogenesis by examining some of the inflammatory cascades that it induces. The experimental approach used native Hz released in the culture medium through parasite schizogony that was collected by magnetic isolation. Using in vitro controlled human infections, it was demonstrated that the relationship between Hz and inflammation as indicated by cytokine production occurs even a few hours upon the introduction of Hz. Hz promotes the production of several cytokines and chemokines, in vitro, early after stimulation in agreement with plasma cytokine levels in CM, also described by several previous studies [27, 45–47]. Hz has been demonstrated to carry malarial DNA, which contributes towards the high inflammatory activities of the immune system during malaria infection [48, 49]. This DNA associated with the Hz may also be responsible for the inflammatory activity that was observed. Furthermore, both natural and synthetic Hz have been reported to induce inflammation both in vitro and in vivo [27, 50, 51]. Thus, data support the hypothesis that this parasite metabolite is likely to play a key role in malaria immunopathology possibly through immunostimulatory activities.

The acute SMA cases had high percentages of circulating Hz-loaded monocytes accompanied by poor TNF and IL-6 production. Phagocytosed haemozoin has been previously shown to inhibit monocyte functions related to immunity [29, 52] and this is in line with the results of this study. Furthermore, the poor activation of monocytes that affected cytokine production may also be due to other schizogony-associated debris from the host and parasite by immune cells, as shown previously [43]. The presence of haemozoin in phagocytic cells is confirmed by microscopy of these cells in the brain tissue, placenta and peripheral blood [53]. This suggests that Hz may also be implicated in malaria-related cell lethargy, which is linked to immune dysfunction [34–38].

The reduction in the expression of co-stimulatory molecules such as CD83, CD80 and CD1a, important for antigen presentation of T cells during malaria infection, has been shown before [52, 54]. These results expand on these data to show additional primary markers on antigen-presenting cells of HLA-DR and CD86 decrease at some point during an acute malaria episode but normalize during convalescence. Schizogony is known to trigger a transient increase in the numbers and movement of peripheral phagocytes [55], and their increased phagocytic activity and uptake of Hz may contribute to subsequent immune imbalance. Microscopical analysis of peripheral blood phagocytes in this study reveals a relatively rapid uptake of Hz upon stimulation accompanied by a significant increase of inflammatory cytokines.

Contrary to one previous report [56] but similar to the findings of others [60, 61], serum levels of IFN-γ were high in CM patients compared to healthy controls. This could be due to geographical, host, and parasite strain differences. However, as previously observed with monocytes [46], there was an impaired function in the T cells of the CM by their limited production of IFN-γ upon stimulation with schizonts lysate. The production of IFN-γ is central to controlling *Plasmodium* infection in both the liver and blood stages [57]. However, while the T-cells appear to be minimally impacted by the malaria infection and disease status in their ability to produce certain cytokines, the ineffective upregulation and expression of adhesion and antigen-presentation molecules by the phagocytes in the bloodstream suggest that the modulation of these cells by Hz could impact the maintenance of a protective immune response. IFN-γ is produced by both CD4 + and CD8 + T cells and other immune cells such that the early appearance of IFN-γ after infection correlate with rapid parasite clearance thereby conferring some protection against the development of clinical malaria symptoms in humans [58] and in mice [59].

As demonstrated in previous studies [60, 61], serum levels of both pro-inflammatory and anti-inflammatory cytokines were significantly higher in children presenting with all clinical forms of malaria than in healthy controls. These observations indicate that acute malaria, regardless of severity, is characterized by higher-than-normal levels of a broad range of cytokines. Cytokines have a short life in plasma with a time range estimated to be between 4 to 12 h [62]. However, CM pathogenesis is a progressive process and as such, the pro-inflammatory indicators likely increase with time. Therefore, sampling over time may allow the trace of disease severity and the role that cytokines play at the different stages of the infection.

The CM group had significantly elevated levels of IL-6, TNF, and IL-10 compared to the controls. Similarly, as has been shown previously, there were higher than normal levels of the predominantly anti-inflammatory cytokine IL-10 in the CM group compared to levels observed in healthy controls and in the other clinical malaria controls. Furthermore, IL-10, which has previously been assumed to play a protective role in Kenyan Children against SMA [15], and confers protective levels with age [3], inhibited TNF production in a dose-dependent manner. The protective role of IL-10 and other anti-inflammatory cytokines such as IL-13 and TGF-β to prevent tissue damage by preventing excess inflammation by downregulating the pathogenic effects of pro-inflammatory cytokines, such as TNF and IL-6, is well defined [20, 43, 63].

The observation that serum cytokine levels were higher in CM cases while the proportion of cytokine-producing monocytes was low does pose an interesting paradox, which has previously been noted [43]. One possible explanation is the high serum cytokine levels are produced by cells other than monocytes (or macrophages), possibly NK cells. In addition, there is some lymphopenia among children with cerebral and uncomplicated malaria [64]. Therefore, elevated cytokine production by lymphocytes in these groups would either come counterintuitively from peripheral blood lymphocytes, or from lymphocytes retained in secondary lymphoid tissues or sequestered in other vascular structures. Ideally, longitudinal studies involving controlled inoculation of the malaria parasites need to be conducted in order to explain this paradox adequately [65].

## Limitations

This study had several limitations; the known limitation of using immunoassays to measure ex vivo cytokine levels is the short half-life of cytokines in plasma. Therefore, these data are regarded as minimal estimates of pro- and anti-inflammatory cytokine concentrations. Secondly, the functional experiments were conducted on blood samples collected at two time points during acute disease and once in convalescence, approximately 30 days post-treatment. Conducting a longitudinal study in which children presenting with different forms of malaria are recruited and then followed closely to provide a time course curve for these cytokines functional studies could provide greater resolution. Such an approach has been attempted before with venous blood samples from South African adults [39], who unfortunately were only followed for 5 days. Thirdly, an unavoidable limitation of clinical studies of natural infection is that neither the time of *Plasmodium* sporozoite inoculation by the mosquito nor the time when merozoites first emerge from the liver to invade erythrocytes is known. Such prior knowledge would be useful in determining the course of the infection.

## Conclusion

In concert, these findings stress the significance of Hz-mediated impairment of monocyte function as both cytokine-producing cells, and antigen-presenting cells. Hz is a potent inducer of pro-inflammatory cytokines and chemokines in vivo. Although a predominantly pro-inflammatory response is essential for clearing parasitaemia during the early stages of the infection, this study suggests that, as the disease progresses, there is an increased role of regulatory cytokines such as IL-10 in suppressing the production of pro-inflammatory cytokines.

## Supplementary Information

**Additional file 1: Figure S1**. Gating strategy for cytokine producing monocytes: Whole blood samples were stimulated with LPS, labelled with CD14 APC, lysed with 2.0ml of 1 x FACS lysing solution and fixed with BFA before the labelling with Isotype Control (PE) and various cytokine antibodies (PE). Flow cytometer dot plots illustrating the side scatter plot versus CD14 (A) with R1 gate for CD14+ cells (monocytes), the Isotype Control plot for setting the gates (B), IL-6 producing monocytes (CD14+IL-6+ cells) (C) and TNF-α producing monocytes (CD14+TNF-α+ cells) (D).**Figure. S2** Gating strategy for monocytes expressing HLA-DR and CD86: Whole blood samples were stimulated with LPS, labelled with CD14 APC, HLA-DR-FITC and CD86-FITC and incubated for 20 minutes. The samples were then lysed with 2.0ml of 1 x FACS lysing solution and washed with PBS before acquisition on Flow Cytometer. The dot plots (A) illustrate the side scatter plot versus CD14 (A) with R1 gate for CD14+ cells (monocytes), geometric mean florescence intensity (GMFI) of HLA-DR (B) and CD86 (C) expression on monocytes from children presenting with different malaria clinical types** Figure. S3** Gating strategy for cytokine producing T cells: Whole blood samples were stimulated with PMA+ION, labelled with CD3 PerCP, lysed with 2.0ml of 1 x FACS lysing solution and fixed with BFA before the labelling with Isotype Control (PE) and various cytokine antibodies (PE). The Flow cytometer dot plots illustrate the side scatter plot versus CD3-PerCP (A) with R1 gate for CD3+ lymphocytes (Total T cells), the Isotype Control plot for setting the gates (B), INF-γproducing cells (CD3+IFN-γ+ cells) (C) and TNF producing T cells (CD3+TNF+ cells) (D).

**Additional file 2: Table S1**. Clinical characteristic of children with cerebral malaria versus the healthy controls in the second paediatric cohort recruited between 2013 and 2016.

## Data Availability

Not applicable.
