# Correction: The ability of Interleukin–10 to negate haemozoin-related pro-inflammatory effects has the potential to restore impaired macrophage function associated with malaria infection

**DOI:** 10.1186/s12936-023-04643-x

**Published:** 2023-07-20

**Authors:** Dumizulu Tembo, Visopo Harawa, Tam C. Tran, Louise Afran, Malcolm E. Molyneux, Terrie E. Taylor, Karl B. Seydel, Tonney Nyirenda, David G. Russell, Wilson Mandala

**Affiliations:** 1 Malawi-Liverpool-Wellcome Trust Clinical Research Programme, Blantyre, Malawi; 2 Department of Microbiology and Immunology, College of Veterinary Medicine, Cornell University, Ithaca, NY USA; 3 Liverpool School of Tropical Medicine, Liverpool, UK; 4 University of Liverpool, Liverpool, UK; 5 Blantyre Malaria Project, Blantyre, Malawi; 6 Michigan State University, Michigan, USA; 7 Kamuzu University of Health Sciences, Blantyre, Malawi; 8 Acadamey of Medical Sciences, Malawi University of Science and Technology, Blantyre, Malawi

**Correction: Malaria Journal (2023) 22:125** **https://doi.org/10.1186/s12936-023-04539-w**

Following publication of the original article [1], it was brought to the authors' attention that there was an error in panel A of Fig. 3: the purple lines in the graphs had been rendered in blue while the blue ones had been rendered in purple. This formatting error has since been corrected in the published article and the corrected Fig. 3 may be seen in this erratum for reference.

**Fig. 3:**
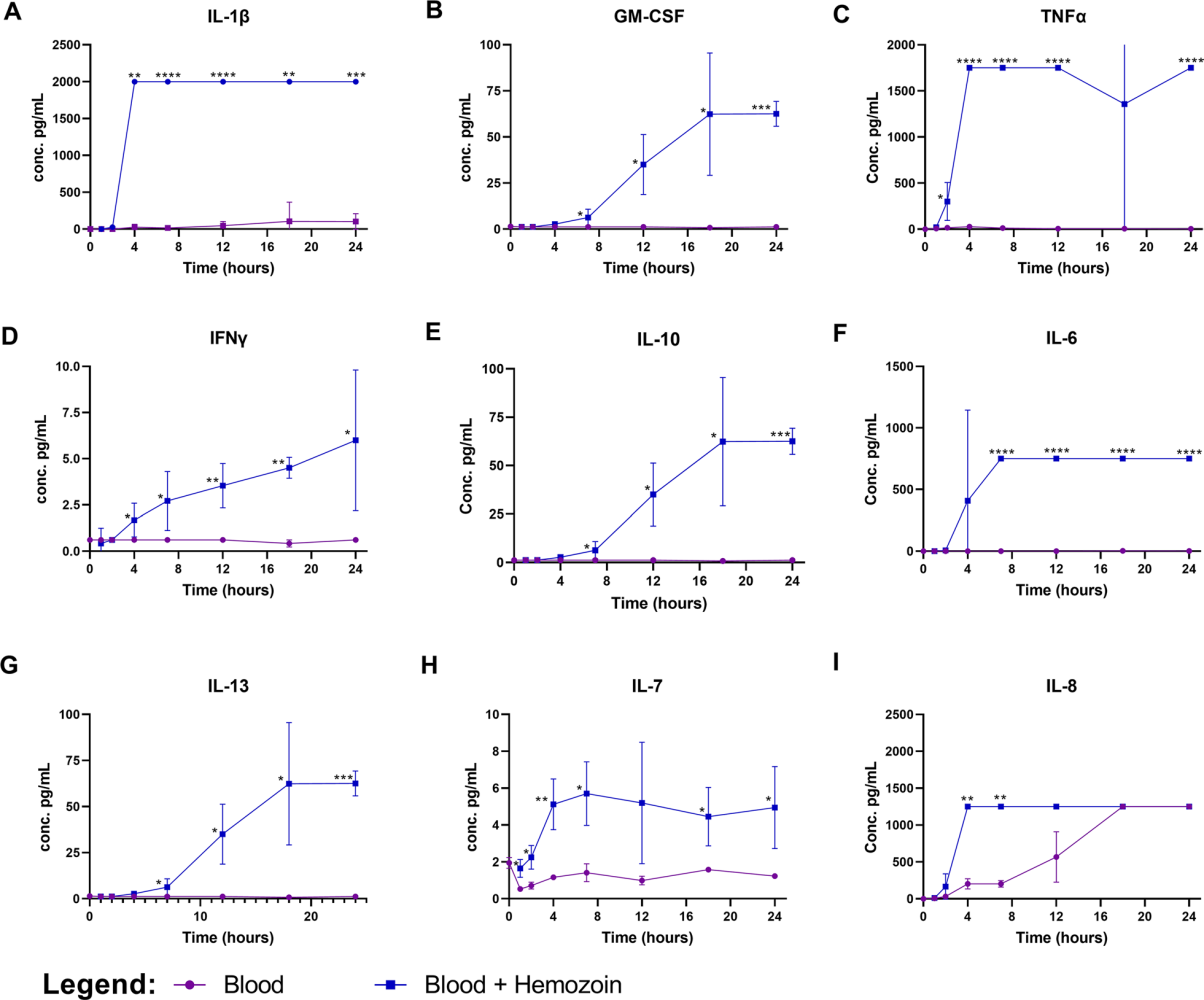
The effect of haemozoin on cytokine production in vitro: Diluted whole blood from healthy volunteers were stimulated with haemozoin at a fnal concentration of 60 nmol/mL at 37 °C. Supernatants were collected at 4, 8, 12, 16, 20 and 24 h. Cytokines IL-1β (**A**), GM-CSF (**B**), TNF (**C**), IFN-γ (**D**), IL-10 (**E**), IL-6 (**F**), IL-13 (**G**), IL-7 (**H**), and IL8 (**I**) are measured over time. The 95% confdence interval for each cytokine is reported. Asterisks show signifcant diferences found between unstimulated blood (purple) and blood stimulated with haemozoin (blue) with multiple comparison t-test. *, p ≤ 0.05; **, p ≤ 0.001; ***, p ≤ 0.0001; ****, p < 0.0001

The authors thank you for reading this erratum and apologize for any inconvenience caused.

## References

[1] Tembo D, Harawa V, Tran TC, Afran L, Molyneux ME, Taylor TE, Seydel KB, Nyirenda T, Russell DG, Mandala W (2023). The ability of Interleukin–10 to negate haemozoin-related pro-inflammatory effects has the potential to restore impaired macrophage function associated with malaria infection. Malar J.

