# Supplementary figures and images for: The ability of Interleukin–10 to negate haemozoin-related pro-inflammatory effects has the potential to restore impaired macrophage function associated with malaria infection
Source: Malar J. 2023 Apr 14;22:125. doi: 10.1186/s12936-023-04539-w (PMC10103463; doi:10.1186/s12936-023-04539-w)

Figure S1

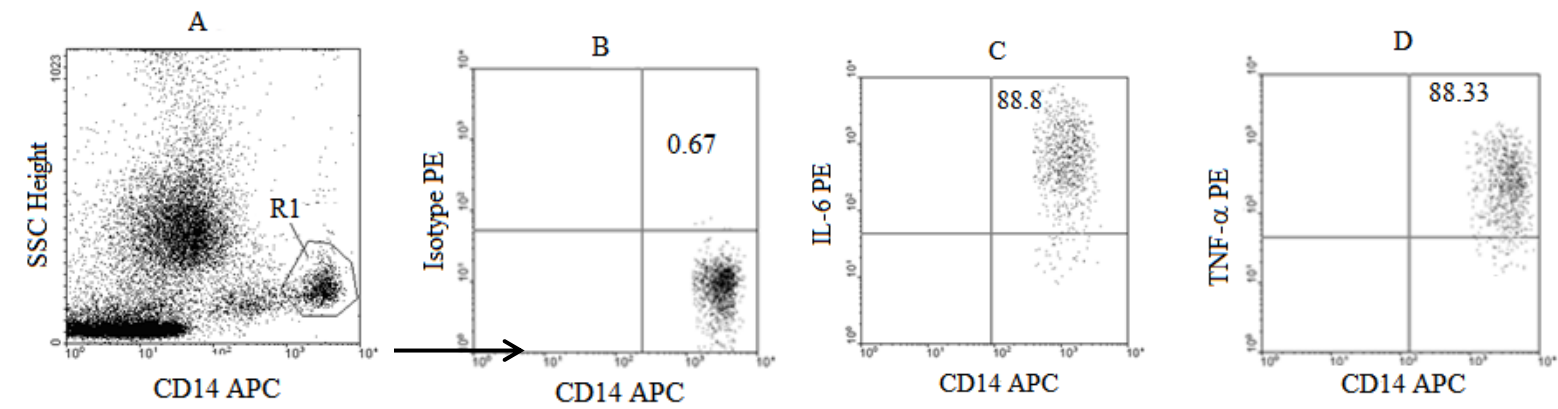

Figure S2

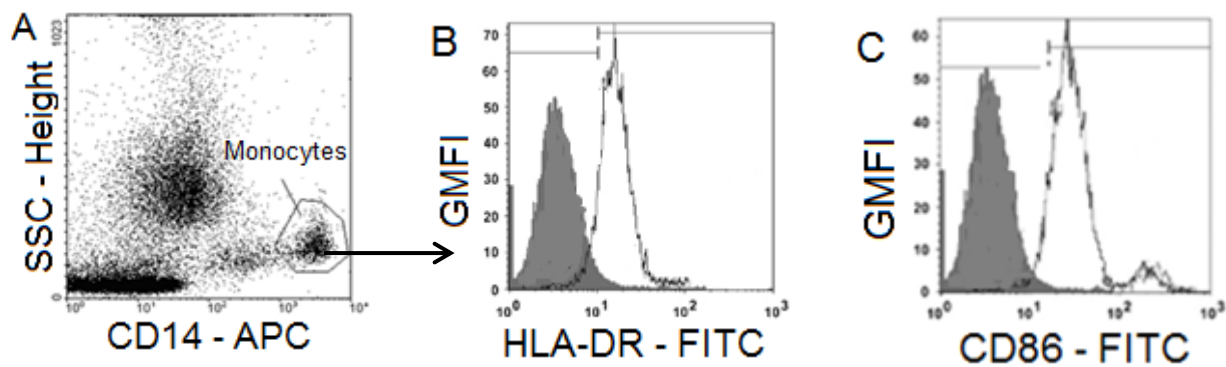

Figure S3

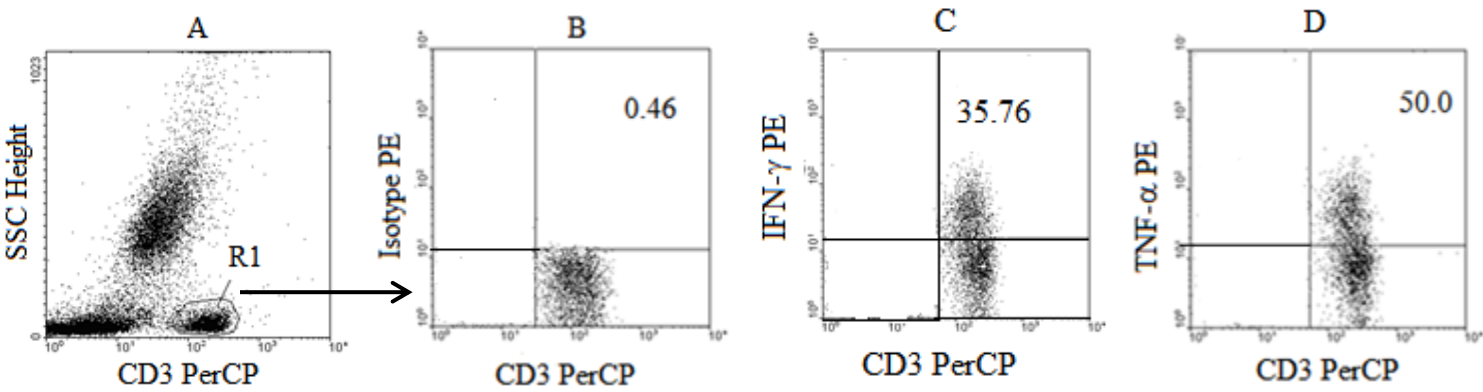

Supplement: Supplementary file 1 — Additional file 1: Figure S1. Gating strategy for cytokine producing monocytes: Whole blood samples were stimulated with LPS, labelled with CD14 APC, lysed with 2.0ml of 1 x FACS lysing solution and fixed with BFA before the labelling with Isotype Control (PE) and various cytokine antibodies (PE). Flow cytometer dot plots illustrating the side scatter plot versus CD14 (A) with R1 gate for CD14+ cells (monocytes), the Isotype Control plot for setting the gates (B), IL-6 producing monocytes (CD14+IL-6+ cells) (C) and TNF-α producing monocytes (CD14+TNF-α+ cells) (D).Figure. S2 Gating strategy for monocytes expressing HLA-DR and CD86: Whole blood samples were stimulated with LPS, labelled with CD14 APC, HLA-DR-FITC and CD86-FITC and incubated for 20 minutes. The samples were then lysed with 2.0ml of 1 x FACS lysing solution and washed with PBS before acquisition on Flow Cytometer. The dot plots (A) illustrate the side scatter plot versus CD14 (A) with R1 gate for CD14+ cells (monocytes), geometric mean florescence intensity (GMFI) of HLA-DR (B) and CD86 (C) expression on monocytes from children presenting with different malaria clinical types Figure. S3 Gating strategy for cytokine producing T cells: Whole blood samples were stimulated with PMA+ION, labelled with CD3 PerCP, lysed with 2.0ml of 1 x FACS lysing solution and fixed with BFA before the labelling with Isotype Control (PE) and various cytokine antibodies (PE). The Flow cytometer dot plots illustrate the side scatter plot versus CD3-PerCP (A) with R1 gate for CD3+ lymphocytes (Total T cells), the Isotype Control plot for setting the gates (B), INF-γproducing cells (CD3+IFN-γ+ cells) (C) and TNF producing T cells (CD3+TNF+ cells) (D). [file 12936_2023_4539_MOESM1_ESM.pdf]
