# Supplementary material for: The ability of Interleukin–10 to negate haemozoin-related pro-inflammatory effects has the potential to restore impaired macrophage function associated with malaria infection
Source: Malar J. 2023 Apr 14;22:125. doi: 10.1186/s12936-023-04539-w (PMC10103463; doi:10.1186/s12936-023-04539-w)
Supplement: Supplementary file 2 — Additional file 2: Table S1. Clinical characteristic of children with cerebral malaria versus the healthy controls in the second paediatric cohort recruited between 2013 and 2016. [file 12936_2023_4539_MOESM2_ESM.docx]

**Table S1:** Clinical characteristic of children with cerebral malaria versus the healthy controls in the second paediatric cohort recruited between 2013 and 2016.

| **Characteristic** | CM, n=54 | Controls, n=40 | P value |
| --- | --- | --- | --- |
| **Age (months)** | 51.15±31.48 | 2.737 ±1.28 | <0.001 |
| **Sex (% male)** | 50 | 40 |  |
| **Temperature (^o^C)** | 38.77±1.18 | NA |  |
| **Respirations (breaths/min)** | 41.11±10.79 | NA |  |
| **Pulse (beats/min)** | 147.6±24.06 | 107.0 ±10.58 | <0.001 |
| **Parasitemia (parasite/μL)** | 171847±314174.2 | NA |  |
| **Hematocrit (%)** | 22.5±7.12 | 34.22±3.62 | <0.001 |
| **Neurosequelae (%)** | NA | NA |  |
| **Death (%)** | 8.00 | 0 |  |

Kruskal Wallis test was used to compare continuous variables and the dichotomous variables were compared using χ^2^ test. The values are reported as means with their standard deviation. The dichotomous variables are reported as percentages. *P* value is considered significant if <0.05.

**Table S2:** Product numbers and clones of the monoclonal antibodies used in the study

|  | **Antibody** | **Product Number** | **Clone** | **Supplier** |
| --- | --- | --- | --- | --- |
| 1 | Anti-CD3-PerCP | 347344 | SK7 | BD |
| 2 | Anti-CD4-FITC | 340422 | L120 | BD |
| 3 | Anti-CD8-FITC | 347313 | SK1 | BD |
| 4 | Anti-CD14-APC | 340436 | MΦP9 | BD |
| 5 | Anti-CD86 | 555657 | 2331(FUN-1) | BD Pharmingen |
| 6 | Anti-HLA-DR | 347363 | L243 | BD |
| 7 | Anti-TNF-α-PE | 340512 | 6401.111 | BD FastImmune |
| 8 | Anti-IFN-γ-PE | 340452 | 25723.11 | BD FastImmune |
| 9 | Anti-IL-6-PE | 554545 | MQ2-13A5 | BD Pharmingen |

1. BD = Becton Dickinson.
2. Phycoerythrin (PE)
3. Peridinin chlorophyll protein (PerCP)
4. Allophycocyanin (APC)
